# Disruptive business value models in the digital era

**DOI:** 10.1186/s13731-022-00252-1

**Published:** 2023-01-16

**Authors:** Navitha Singh Sewpersadh

**Affiliations:** College of Accounting, University of Cape Town (UCT), 4th Floor, Leslie Commerce, Rondebosch, Cape Town, 7701 South Africa

**Keywords:** Artificial intelligence, Bots, Business model, Customer relationship, Digital transformation, Innovation, Networks, Services, Technology, Value drivers

## Abstract

The coronavirus pandemic illustrated how rapidly the global environment could be disrupted on many levels but also drive an acceleration in others. Business leaders are grappling with dysfunctional business models that are ill-equipped to manage the disruptive environment of growing artificial intelligence. Hence, this study examined the discontinuous shift in the scope and culture of business models by exploring interdisciplinary streams of literature. An integrative review methodology was used in this study to develop theoretical constructs relating to business model innovation in the services sector. Key propositions were an innovation continuum, a responsive business innovation model and value architecture, which inculcates a sustainable value creation proposition and market advantage. Businesses must continuously evolve on the high end of the innovation continuum to reduce the risk of innovation apathy and strategic myopia. A key contribution of this study was the interdependencies in value networks that allow for collaborative working and co-creation of resources, such as crowdsourcing, crowdworking and social media platforms. This study also showed the growing importance of a centre of excellence to function at the forefront of disruptive technologies. A key finding was the need for governance structures to recognise and manage the trade-offs between value drivers, which sometimes may conflict with societal benefits. The integrative review revealed that customer relationship management, global business services and artificial intelligence had not been unified in the extant literature, which makes this paper novel in its contribution to businesses struggling with or opposed to the digital revolution.

## Introduction

The evolution of technology has disrupted almost every business globally by continuously transforming, enhancing, and streamlining operational processes and procedures. Digitalisation^1^ is disruptive and brings about discontinuous changes (Paiola & Gebauer, 2020), but it is a key element for new value-creation and revenue-generation opportunities for market competitiveness (Kamalaldin et al., 2020). Climate change, pandemics, environmental devastation and widening social inequalities have created an abrupt realisation that the existing business models are no longer ‘fit for purpose’. New practices, skills, operational processes, and business models are required to use artificial intelligence^2^ (AI) to create value for customers (Sjödin et al., 2021). It is increasingly important for businesses to understand the evolving environment to assimilate for viability in the market and then innovate to gain a competitive advantage. Businesses face pressure to focus on achieving their non-financial goals and not just maximising profits (Rabaya & Saleh, 2022). The interconnected elements of environmental, societal and governance (ESG) have provided a catalyst to transform businesses to be more responsive toward the planet and people when pursuing profitability and growth. *“The illiterate of the twenty-first century will not be those that cannot read or write, but those that cannot learn, unlearn and relearn”* (Toffler, 1970). Refining, adapting, revising and reformulating a business model provides businesses with a roadmap for achieving holistic goals by harnessing the strategic advantages of AI technologies.

Digital transformations create new potential for organisations to redefine and optimise their operations by recognising the role of automation^3^ in creating market differentiation and service excellence (Flyverbom et al., 2019; Zuboff, 1988). The COVID-19 pandemic affected critical business functions across organisations globally, thus serving as an accelerator of digital transformations and the reconfiguration of static business models. The pandemic affected how people operate and customer services are provided, particularly when governments imposed regulated lockdowns to protect human life. According to institutional theory, internal and external pressures (Zucker, 1987) accelerate the desire or compulsion to transform an organisation. One such pressure is disruptive digital technology, and the other is the pandemic. The traditional workforce has also been transformed into a blend of humans working collaboratively with AI.

A global survey conducted by Deloitte (2020) found that the largest concern for respondents during the pandemic was the viability of their business models. Some businesses led the business model innovation^4^, while other companies crumbled. As the contingency theory proposes (Lewin & Volberda, 1999), a suitable strategy is required to accomplish a strategic fit with an organisation’s market. Therefore, business model innovation is a key ingredient in underpinning a business resilience strategy, particularly with technological innovation rapidly changing the nature of work. These pressures to innovate in the digital era have widened the gap between innovators and stragglers in the business world. The advantages of conventional business processes that are human reliant are weakening, exposing the fragility of the human capital leverage model, which will be further impacted as AI evolves. Therefore, innovation laggards may fail should they not embrace the principle of accelerating disruptive technologies in their business models. As global economies face unprecedented disruption, a once disruptive business model can become static by becoming complacent or relying excessively on past strategies that may have become outdated. This risk of innovation apathy or myopia motivates businesses to have an agile business model that continually evolves with the disruptive digital era.

A business model is seen as a robust abstract instrument to model a framework for a company’s competitive stance (Hamel, 2000) by connecting technical potential with the recognition of economic value (Chesbrough, 2011). However, Teece (2010) argued that approaches to business models are diverse due to the absence of a theoretical grounding in economics or business studies. For this reason, there have been calls for research on business models and value propositions^5^ focusing on market differentiation and industry disruption (Weinstein, 2020). Emerging market differentiators are concentrated on labour automation, such as Robotic Process Automation (RPA) and service bots used in Global Business Services (GBS) (OECD, 2007; SSON, 2018). However, codifiability and digitalisation in the global services literature are absent despite the advantages of the centrality of transaction costs and efficiencies (McWilliam et al., 2019). There is an ongoing call for researchers to adapt and extend how AI technologies can be aligned with business (Coltman et al., 2015; Santos et al., 2020; World Trade Organization, 2019). Moreover, a persistent gap exists in academic research regarding the business models using AI for digitalising Customer Relationship Management (CRM) in the global service sector. A necessary first step toward knowledge evolution and model building is a systematic exposition based on theory (Melville et al., 2004) and disruptive technology (Parmar et al., 2014) that drive an understanding of business model innovation (Teece, 2018) to capitalise on business opportunities that overcome pandemic challenges.

With digital servitisation^6^ (Kohtamäki, et al., 2019; Vendrell-Herrero et al., 2017), the service sector is no longer operating as a separate category, since retailers and manufacturers are entering the service sector with smart services, such as Caterpillar, Michelin, Siemens and Voith Group. They transform their products by embedding software to communicate to the data cloud (Ng & Wakenshaw, 2017), which can then be analysed through advanced data analytics for co-created value-added services (Opresnik & Taisch, 2015). This study selected the service sector to examine business model innovation, since it is people-centred and an important contributor to the economic environment. A GBS structure was adopted in this study, because it allows the researcher flexibility to incorporate innovative systems with global mobility for the service sector’s offerings. The GBS business model also provides benefits of economies of scale, streamlined processes, superior service quality and scalability of operations through consolidating support functions into a single centre staffed with specialists. This article provides crucial theoretical framing by linking the CRM, GBS and service innovation technologies to business model innovation. This study contributes an innovation continuum, a responsive business innovation model and value propositions focused on market differentiation, service innovation and industry disruption. This study also provided a research agenda to catalyse future research.

## Research methodology

This study employed a methodical means of assembling and synthesising previous research (Baumeister & Leary, 1997; Tranfield et al., 2003) through an integrative review process of experimental and non-experimental research with theoretical and empirical data (Whittemore & Knafl, 2005). This study adopted a concept-centric rather than a chronological or author-centric approach (Webster & Watson, 2002) due to the inclusion of four streams of literature: GBS, CRM, service innovation and business models.

**Fig. 1:**
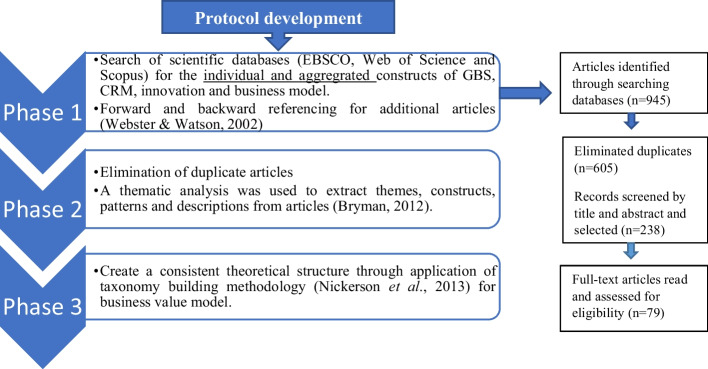
Phases of the integrative review. Source: Author

As Webster and Watson (2002) envisaged, the research process started with a protocol development to create a defined body of literature for the theoretical development of a responsive business innovation model. The protocol had three phases, as depicted in Fig. 1. The first phase mitigated the incompleteness risk of the literature review by systematically identifying and reviewing existing databases. While the second phase remedied the overlap from different databases by filtering for duplicates, the final phase focused on creating a consistent structure among all patterns. There was rigorous screening and appraisal of each paper to assess whether its content was fundamentally relevant. A final sample of 79 high-quality articles was selected to build the theoretical constructs for this study. Other articles published by technology or accounting firms in this paper’s literature review and results section were used to establish current market practices. Whittemore and Knafl (2005) stated that the suppositions of the integrative review could be reported in tabular or diagrammatic form. Since the study intended to develop a theoretical business model in the form of a diagram, a thematic analysis was used to consolidate further and conceptualise higher levels of themes, constructs, patterns and descriptions from articles associated with GBS, CRM, service innovation technologies and business models.

## Literature review

A theoretical framing is required for constructing a response business model. A business model provides a rationale, design or architecture for strategic choices to create, deliver and capture value (Magretta, 2002; Osterwalder & Pigneur, 2010) by specifying the structural elements and technology to address the unmet needs and activities of customers (Teece, 2018). Accordingly, organisational theory (strategic decision-making), customer relationship management (customer needs), global business service (structure) and service innovation technology provide the grounding for this research.

### Organisational theories

The institutional theory provides a multifaceted business outlook on normative pressures from external and internal sources that influence organisational decision-making (Zucker, 1987). It determines conventional rules and assumptions (Oliver, 1997), whereby conformance to these norms is compensated through improved legitimacy, resources and survival capabilities (Scott, 1987). Institutions provide social structures, rules and resources that are important to the service sector. Adopting AI in the service sector differentiates the fourth industrial revolution from the third (Schwab, 2017), which triggers adaptive structural processes that progressively change the organisation’s social interaction rules and resources that determine decision efficiency outcomes (DeSanctis & Poole, 1994). In the knowledge economy^7^ (Powell & Snellman, 2004), greater reliance is placed on the intellectual capabilities of intangible resources as opposed to physical resources for decision-efficiency outcomes.

Extrapolating these theories to the fourth industrial revolution, it is apparent that there are challenges that organisations face to conform to the normative pressures of digital disruption that depend upon each company’s specific circumstances (contingencies). “*A good business model begins with an insight into human motivations and ends in a rich stream of profits*” (Magretta, 2002 pg. 3). Each organisation needs to find a strategic fit within the knowledge economy to gain value-driving opportunities while accelerating its customer-centric initiatives. For this reason, the customer relationship management (CRM) literature provides a framework to delve into human motivations concerning their buying incentives, biases and emotional connections.

### Customer relationship management

The core of CRM is understanding customer needs and leveraging that knowledge to increase a firm’s long-term profitability (Stringfellow et al., 2004). In the digital era, technology may be leveraged to be customer focused to understand customer needs better. For instance, probing large data sets (big data) may inform CRM strategies (Payne & Frow, 2005; Stringfellow et al., 2004). Customer data is a rich source of unstructured, voluminous and ambiguous data for further processing through analytics. Data analytics are recommended for managerial strategic decision-making, since it is grounded in evidence rather than perception (IBA Global Employment Institute, 2017; McAfee, et al., 2012). Knowledge gained from data analytics is essential for building close customer relationships for service differentiation, customer loyalty and value creation.

Irrespective of the industry, the desire to nurture customers is a key success factor driving the need for CRM differentiators to gain a strategic competitive advantage. However, Stringfellow et al. (2004) criticised knowledge-deficient models developed from superficial customer data (demographics and transactions), since these do not address the functional (purpose-fulfilling) and emotional requirements of customers. They used the study by Schneider and Bowen (1999) to illustrate that decision-making is not dictated by functional needs, since a man may pay double the price to buy a Ralph Lauren polo shirt instead of a similar unbranded polo shirt to fulfil his self-esteem needs. This diversity in customer decision-making illustrates that relational selling may sometimes outweigh value-based selling. Therefore, any customer-centric business model should understand that buyers are not always rational but emotionally guided. For this reason, sales or services can be categorised as value-based to fulfil purpose or relational to fulfil the emotional connections to the product or service.

### Global business services

According to OECD (2007), business services are provided to other businesses instead of customers. Organisations wanting to reduce costs enter the outsourcing market for lower-cost business services. However, within a GBS, various processes and functions are shared and operate unitedly instead of using several shared service centres and dealing with outsourcing vendors independently. The principal objective of GBS is to provide business-to-business services at a reduced fee and at contracted levels of quality that improve practice through lean, cost-competitive, efficient and streamlined processes with an optimised cost structure (Daub et al., 2017; OECD, 2007; SSON, 2018). This goal is achieved by leveraging a range of enablers, including a robust customer interaction framework, standardisation, economies of scale, automation, organisational realignment, labour/robotic arbitrage, implementation of best practices and true “end-to-end” process optimisation (SSON, 2018). Thus, companies leverage a GBS model to gain market advantage and operational efficiencies through an agile, focused and leaner service organisation. GBS integrates services that forsake functional silos and transcends to a multifunctional collaborative approach. GBS has an amalgamated delivery model providing “back-office” services to a global customer base, such as accounting, finance, HR, IT and procurement, and increasingly moving to “front office” activities, such as sales, marketing, analytics and reporting (SSON, 2018). Currently, businesses are focussed on services related to their digital offerings and the analytics of their customers’ data. Geographical expansion, innovation quest and the adoption of new technologies are important in pursuing profits when competition is rife (Hodgson, 2003). GBS, with AI technology, has an opportunity to achieve scalability by integrating its multitude of centres into a single network to expand its range of business across the globe for a competitive advantage.

Most GBS users depend heavily upon intangible assets, particularly technological and service innovations (OECD, 2007). GBS centres can integrate automation, virtualisation and analytics, amongst other digital tools and capabilities, into their prevailing processes that provide more effective support to business units (Daub et al., 2017). Global organisations, such as Siemens, have incorporated a GBS-type structure into their global multifunctional business model that provides shared services to all Siemens businesses. The two fundamental principles that guide this organisation’s international services centres are customer satisfaction and continuous improvement through innovation (Siemens, 2020). For this reason, the GBS-type structure has extended to accounting firms, with their large global networks increasingly centralising certain remote auditing functions through technology and then outsourcing geographic-dependent work to their component auditors. For the longevity of any business, new organisational designs need to evolve that shape human workers, such as service innovation technologies.

### Service innovation technologies

The innovation theory proposes that innovations diffuse from early adoption to widespread use (Rogers, 1995). However, innovations have a lag effect on their relative advantage (profitability, social prestige, other benefits) over its predecessor. In defining a technology readiness index ranging from innovators to laggards, Rogers (1995) elaborated on the speed of the adoption being positively related to the perceived benefits, compatibility with the company’s structures, ease of use and trialability (experimental capability). The innovation diffuses at the rate at which an innovation’s results are visible to others (observability). However, the complexity of the innovation is negatively related to the speed of the adoption. Understanding innovation theory is central to constructing or transforming a business model.

The quadruple-helix theory proposes that society can drive the innovation process to design sustainable strategies to achieve social innovations in a green economy (Carayannis et al., 2012, 2020). ESG goals are increasingly being demanded by stakeholders to be incorporated into business models. The focus on ESG has led to traditional business models integrating sustainability while undergoing digital transformation. A sustainable business model delivers multifaceted value to a wider range of stakeholders when compared to the traditional business model (Bocken, et al., 2013). Digital technologies allow for strategic planning on economic, social, and environmental performance (Evans, et al., 2017). For instance, social network platforms may assist companies in achieving their ESG goals allowing companies to move closer to a green economy. Platforms are technologies that facilitate networking for companies to co-create with stakeholders (Allen, et al., 2009). A concept is drawn from the microworking philosophy (Howe, 2008), where a large dynamic network enables the organisation to connect with the internal and external environment for co-creation opportunities. Close company–customer collaboration allows for long-term value co-creation (Kamalaldin, et al., 2020), where customers co-produce services by providing insights. Types of co-creation opportunities are the wisdom of crowds^8^ (Surowiecki, 2004), open innovation^9^ (Chesbrough, 2003), crowdsourcing^10^ (Howe, 2008) and crowdworking^11^ (Ross, 2010). A common feature of these co-creation opportunities is that they all use an open call for knowledge to create innovative solutions. Amazon Mechanical Turk and Uber are examples of the crowdworking philosophy using digital platforms to build networks in the service sector. Leveraging society’s connectivity and responsiveness through platforms facilitates the collaborative designing of personalised products, services and experiences.

Technologies such as RPA and service bots have been widely adopted in the service industry. RPA interacts with the user interface of other computer systems using rule/logic-driven software robots (softbots) that are coded to execute a high volume of repetitive tasks without compromising the underlying IT infrastructure (Deloitte, 2018; van der Aalst et al., 2018; Willcocks et al., 2015). This technology dates to the Eliza programme’s interactive bots that enabled interaction between humans and machines using text-based communication (known as the Turing test) (Turing, 1950; Weizenbaum 1966). RPA follows prescribed protocols and procedures that increase the speed, accuracy, compliance and productivity of business processes.^12^ Instead of multiple ERP solutions (taking data from one system and inputting it into another system), it is more cost-effective and efficient to integrate RPA into a company’s existing infrastructure and automate processes (van der Aalst et al., 2018). However, RPA is on the lower end of intelligent automation, since it uses structured logic and inputs to operate from simple to complex business tasks.

RPA with cognitive automation has allowed softbots to be more useful due to their superior intelligence. Softbots with machine learning^13^ capabilities are designed to mimic human thought and action to manage and analyse big data with greater speed, accuracy and consistency than humans can achieve by leveraging different algorithms and technological approaches (Firstsource, 2019). Algorithms do not produce definitive solutions but present probability-based predictions for humans to evaluate and make informed decisions. Table 1 provides a summary of the Softbots.

**Table 1:** Softbot functionalities

Functionality	Description	Author
RPA accepts a varied set of sophisticated objectives	Learns new commands, the locations of several objects, and its human partners’ preferences	Etzioni et al. (1993)
RPA technology sits on top of the current IT systems and does not substitute but supplements BPM	No coding experience is required for human users. No need to develop expensive platforms, replace existing applications or manipulate their code, resulting in significantly lower IT investment expenses	Willcocks et al. (2015)
RPA serves as a digital worker and interacts as a human user would with other systems	RPA is granted the same user access rights as given to humans to perform mundane, repetitive processes to reduce the transactional workload of humans. RPA software acts as a digital worker with a login ID and password to perform routine tasks like the HR onboarding process for new employees. RPA releases humans to focus on non-routine HR tasks requiring critical thinking and skills	van der Aalst et al. (2018); Willcocks et al. (2015)
Softbots mainly automate white-collar work, such as accounting, sales, logistics, trading and managerial occupations	Softbots can execute business processes, such as humans typing and clicking in different applications, alternatively taking information from one system and capturing it into another system or analysing documents to gather data	Acemoglu and Restrepo (2019); Agostinelli et al. (2020); Deloitte (2018); Willcocks et al. (2015)

Source: Author

Softbots are also known as service robots, chatbots, AI bots, AI assistants, virtual assistants or agents, and digital assistants or agents. This study adopts the term service robots, since they are most common in customer support or sales environments, where they are expected to serve customers. For instance, call centre jobs are labour-intensive and employing people’ around the clock’ for one or two late-night phone calls are costly. However, service bots can answer simple queries efficiently and far quicker than a person can. Service bots use Natural Language Processing (NLP) to develop logic from unstructured inputs for human interaction. Service bots with NLP, Natural Language Understanding (NLU)^14^ and Natural Language Generation (NLG)^15^ are distinguished from the greater domain of service bots due to their aptitude to employ language to converse with their clients. Table 2 shows the different types of service bots.

**Table 2:** Types of service bots

Type of Service bots	Description	Limitation
NLP or linguistic service bots are rule-based and use if/then logic to generate conversational flows that deliver the fine-tuned control and agility omitted in machine-learning service bots These are the most common bots encountered by the public through live chat, an e-commerce website, or Facebook messenger More advanced service bots are multi-language	Linguistic service bots have a highly labour-intensive approach which can be rigid and slow to develop, since language conditions review the order of words, synonyms and common phrases, ensuring that questions with the same connotation get the same answer Capabilities include interactive, frequently asked questions, delivering specific scheduled communications, slot filling, making reservations, purchasing catalogue items, updating customer profiles, or other basic transactional competencies, such as taking pizza orders Virgin Trains in the UK uses service bots with NLP to automate customer refunds by reading customers’ emails, reducing daily processing time and manual labour by 85 per cent	Interactions with these bots are specific and structured, during which automated tests check the bots’ quality and consistency but cannot correct any bot misinterpretations, since they require humans to modify the conditions
AI service bots use machine learning that is more sophisticated, interactive and personalised than rule-based service bots, since they are more conversational, data-driven and predictive	Algorithms mimic human cognitive functioning allowing service bots to adapt and handle non-standard cases by observing humans resolve problems (such as system errors, unpredicted system behaviour, or changing forms) AI software can sense, reason and act with structured and unstructured data, performing tasks normally associated with human intelligence, such as decision-making, visual/pattern recognition, speech recognition and translation between languages Gradually with the power of data, they become contextually aware and leverage NLU to personalise a user’s experience through predictive intelligence	AI service bots work best if tasks are well matched to their capabilities, since they require a large amount of training data and human training specialists to perform even basic tasks Any malfunctions are difficult to fix, let alone optimise and improve

Source: compiled by Author

Kiat (2017) states that service bots can manage CRM quality by handling mundane tasks leaving salespeople to focus on high-value tasks, such as meeting customers and concluding company sales. In general, leads should be attended to within 5 min to convert them to paying customers, which would be achieved with service bots. Other advantages are:

- **Seamless interface:** bots can recall their previous customer interactions and seamlessly verify customer data by linking to social media, so queries are addressed at a speed unmatched by humans. Service bots can also seamlessly transfer complicated cases to human operators, facilitating humans’ foci on higher value customer engagements.
- **Data enrichment:** cost-effectively resolving data leakage problems, since humans often neglect to record key customer information from the various stages of the customer’s purchase process, whereas a service bot would automatically capture the discussion.

Service bots are key differentiators within the IT industry with improved revenue performance and customer value (customer contentment, service delivery and contact centre performance) (MIT Technology Review, 2018). Service innovation technologies are employed by renowned brands, such as Amazon, Netflix, Starbucks and Spotify, to name a few. Service bots work reliably and accurately around the clock while maintaining the same competence level without being distracted or fatigued. Service bots also do not have inherent limitations, such as becoming ill, going on strike or requiring leave. In 2019, the banking sector achieved operational cost savings of $209 million from employing service bots. Insurance claims management departments had cost savings of $300 million across motor, life, property and health insurance (Juniper Research, 2019). Artificial Solutions (2020) also reported that Shell attained a 40 per cent decrease in call volume to live agents due to their service bots, Emma and Ethan. They answered 97 per cent of questions correctly and resolved 74 per cent of digital dialogues. Similarly, the service bot Laura is digitally transforming Skoda (a Volkswagen Group’s subsidiary), where customers can discuss their vehicle needs and budget with Laura (Artificial Solutions, 2020). Therefore, digitalisation has resulted in customer relationships evolving from transactional to more relational.

## Results: theoretical propositions

Several constructs emerged from the thematic analysis of the integrative review for developing a digital business model, reflected in Table 3.

**Table 3:** Digital business model key constructs

1. Customer insights, tailoring and satisfaction	Amit and Zott (2012); Dehning and Richardson (2002); Dedrick et al., (2003); Iansiti and Lakhani (2020); Kim (2021); Payne & Frow, 2005); Schryen (2013); Sjödin et al., (2021); Stringfellow et al., (2004)
2. Digital business process enhancements	Downes and Nunes (2013); Sjödin et al., (2021)
3. Data competencies and analytics	Gauthier et al., (2018); Parida et al., (2019)
4. Intangible assets	Dedrick et al., (2003); Schryen (2013); van der Aalst and et al., (2018)
5. Workforce capital	Acemoglu and Restrepo (2019); Agostinelli et al., (2020); van der Aalst et al., (2018); Willcocks et al., (2015)
6. Increasing coordination and productivity	Amit and Zott (2001); Iansiti and Lakhani (2020); Visnjic et al., (2017)
7. Performance/financial returns	Amit and Zott (2001); Dedrick et al., (2003); Dehning and Richardson (2002); Kim (2021); Schryen (2013); Teece (2010); van der Aalst et al., (2018)
8. Environmental and societal values	Anderson and Kupp (2008); Bocken et al., (2013); Boons et al., (2013); Evans et al., (2017); Kim (2021); Sanchez and Ricart (2010); Yang et al., (2014)
9. Sustainable competitive advantage	Casadesus-Masanell and Zhu (2013); Kim (2021); Mitchell and Coles (2003); Morris et al., (2005); Yang et al., (2014)
10. a) Creates value b) Co-creating value	Amit and Zott (2001); Aspara et al., (2013); Casadesus-Masanell and Zhu (2013); Chesbrough (2010); Kim (2021); Magretta (2002); Teece, 2010); Visnjic et al., (2017); Paschou et al., (2020)

Source: compiled by Author

**Fig. 2:**
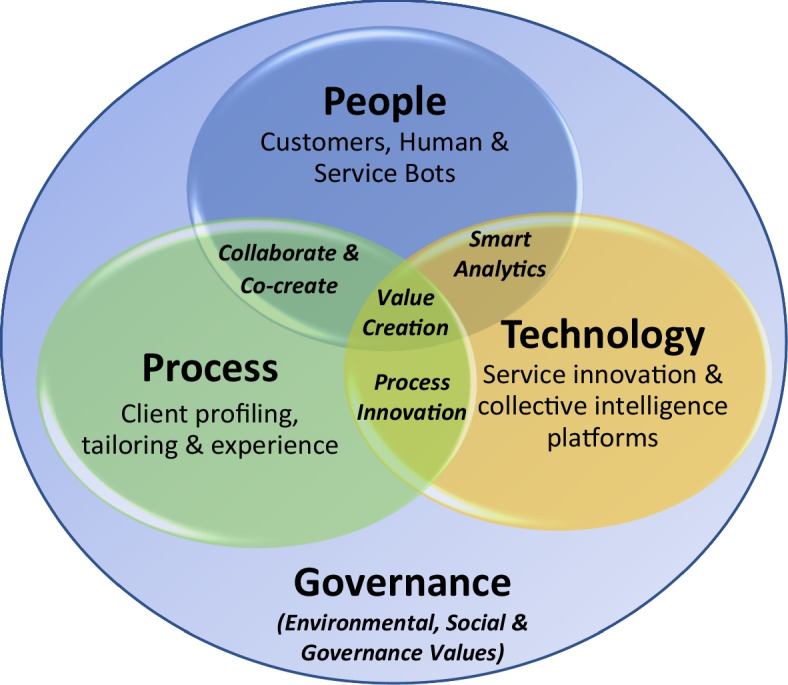
PPTG Framework. Source: Author

Using the people, process and technology (PPT) framework (Leavitt, 1964), these ten constructs from Table 3 and innovation capabilities are presented in Fig. 2. This study has added governance to the PPT framework to form the PPTG framework. Governance is imperative for oversight over the value-creating activities (Sewpersadh, 2019a) to balance the trade-offs from the synergistic benefits of lower costs, increased coordination, greater productivity and value delivery with the ethical and risk concerns over customer data.

In Fig. 2, people have been expanded to include service bots. Collaboration between service bots, employees and customers are integral for value co-creation. Service bots cost-effectively record customer information from the various stages of their service interactions, allowing for data warehousing. Data warehousing is important for allowing data mining tools and the analysis of critical customer parameters.An ethics and risk officer will play a key governance role in overseeing the principles of fairness and ethics over emerging technologies, such as service bots. Increasingly companies integrate their AI technologies with social media platforms which necessitates the ethics and risk officer to detect, correct and prevent any biases that the service bots learn through the data they collect. For example, service bots may discriminate against customers based on their demographics (Puntoni et al., 2021). In 2016, Microsoft launched a service bot called Tay to research conversational understanding. This project failed, because the developers did not anticipate that some Twitter users would teach the bot to make racist, inflammatory and offensive tweets through its Twitter account (Berditchevskaia & Baeck, 2020). For this reason, recent studies proposed digital corporate responsibility to guide ethical dilemmas related to AI technology (Lobschat et al., 2021). There are also ethical and security risks when service bots impersonate humans (van der Aalst et al., 2018), since they may make improper judgements due to contextual changes that may remain undetected, leading to unintended consequences. For instance, service bots may make poor-quality recommendations that do not align with customer interests or may expose customers to vulnerable and risky situations (Mullainathan & Obermeyer, 2017). Service bots require service audits to prevent poor service quality outcomes. Service bots also have excessive access and privileges that place them at risk of cyber-attacks. The ethics and risk officer may assist in safeguarding data using surveillance methods to detect intelligent malware.^16^ Research has found that customers are more likely to act unethically and misbehave (LaMothe & Bobek, 2020) when interacting with service bots. Therefore, service bots need to be monitored to detect and prevent these infringements.

In Fig. 2, PPTG is improved with technologies for process value configuration. Technology with people allows for smart analytics on service value capture and optimisation. For example, service staff, key accounts managers and digital developers in Solutioncorp evaluate customer service data to identify priority areas for AI innovation (Sjödin et al., 2021). This dispersion of emerging technology gives rise to a disruptive landscape in the knowledge economy, necessitating more R&D and continual business model innovation. The three overarching themes from the constructs presented in Table 3 are innovation, sustainable business models and value creation, which will be discussed further below.

### Innovation continuum

**Fig. 3:**
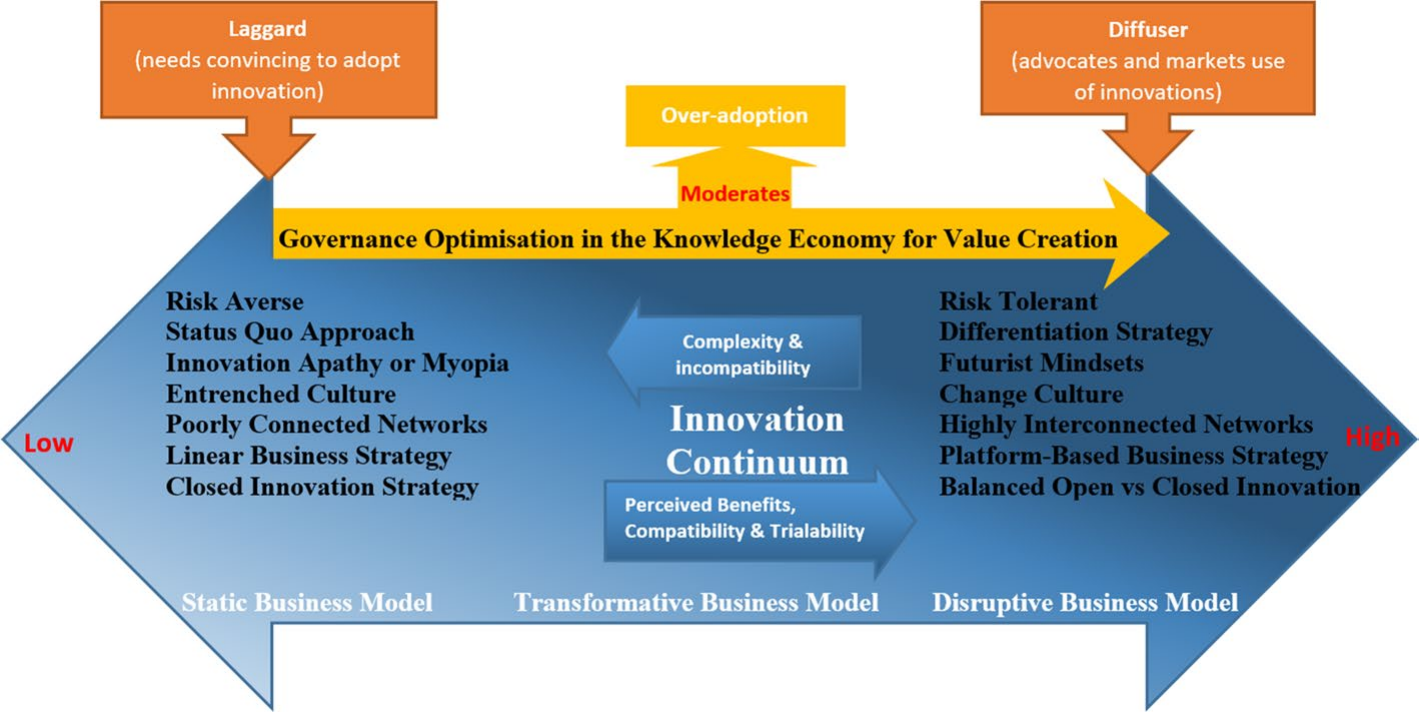
Innovation Continuum. Source: Author

The rapid pace of the evolution in technology innovation accelerates the diffusion of innovations (Rogers, 1995). The increased R&D in innovation creates a continuum (Fig. 3), where companies are not statically classified according to their degree of innovation but rather placed on a continuum. Those businesses that recognise innovations’ relative advantages, compatibility and trialability (Rogers, 1995) will move to the higher end of the continuum. Although, a high-innovation company may not remain a disruptor in the market if it becomes complacent or myopic with its innovation strategy and neglects to continuously improve its business processes. This complacency can be explained by the icarus paradox, where success may lead to a path of convergence with an emphasis on the same strategies, which may simplify and desensitise divergent evolving demands (Elsass, 1993; Miller, 1990). Past successes promote a defensive mindset and overconfidence, resulting in the persistence of the same strategic formulas when executing innovative strategies is the most appropriate response (Sewpersadh, 2019b) to the market’s changing needs. Thus, this paradox may lead to myopia, complacency and inertia. This complacency leads to a condition of ‘unconscious incompetence’, where the lack of knowledge of the availability of advanced technologies leads to suboptimal decision-making or decision paralysis on deploying such technologies. For this reason, the degree of innovation is bidirectional on the innovation continuum, which allows for the acceleration and deceleration of innovation investment. As business models transition from traditional to transformative ones, eventually evolving into disruptive ones, those with myopic capabilities soon find their business models antiquated. When companies intensify their investment in innovation, they adopt a futurist strategy allowing them to transition up the innovation continuum and challenge complacent companies.

Rogers (1995) cautioned that insufficient knowledge, inability to predict consequences or overzealous innovation investments might lead to over-adoption. Also, the complexity or incompatibility of innovations may not be suitable for some businesses, which may jeopardise their positioning on the continuum. For this reason, governance structures, such as a digitalisation committee, are important for moderating the firm’s adoption strategy. This committee will assess the suitability, acceptability, feasibility and sustainability of developing or acquiring innovations. Integrating stakeholder networks in collaborative activities creates trust-based relationships, legitimacy and good governance that allows for the acceptability of innovations. In Fig. 3, governance optimisation is vital for ensuring value-maximising decision-making concerning value-creating activities for all stakeholders (Sewpersadh, 2019a).

There could also be a reluctancy to allocate resources for R&D due to a digital paradox (revenue growth is not as expected despite the proven growth potential) (Gebauer, et al., 2020). For these reasons, value creation and governance optimisation are unidirectional factors in Fig. 3 and are placed on the high end of the continuum, where disruptive business models operate. Governance is essential to moderate the negative effects of an over-adoption, complex or incompatible innovations and the digital paradox. Good governance is also critical for balancing trade-offs when making strategic decisions. For instance, harmonising the need for legally protected intellectual assets for profit maximisation and sustainability with knowledge sharing to build collaborative networks.

Central to the innovation process is the need for firms to create and acquire “new combinations” of knowledge. Based on the resource-based theory, complementary assets and capabilities are scarce but valuable strategic resources, since they have strong path dependencies that are difficult to imitate (Barney, 1991), thus shaping the firm’s competitive advantage in the cooperative network. Since companies compete in a capital-intensive space, with barriers to entry and economies of scale, profits may be achieved with the legal protection of competitive advantages, such as closed innovation. Closed innovation is the internal research within a particular company that is generally protected by patents, so that access to that innovation is controlled by the rightsholder (Chesbrough, 2003). Progressively, open innovation has become a way in which key resources are obtained for the development and execution of innovation (Chesbrough, 2003, 2011). Open innovation is a means of sharing costs, ideas, synergies and skills (Chesbrough & Crowther, 2006) from value networks to co-create innovation rather than an individual company outlaying capital to conduct R&D from scratch. For this reason, in Fig. 3, the networking capabilities of a company also follow the direction of its innovation policy due to the collaborative work with extended networks that allow for the acquisition of external knowledge. As innovation diffuses, collaborators within forged networks stimulate newer co-created innovations with superior outcomes.

A significant limitation to knowledge sharing is the disclosure of internal knowledge to external collaborators (Cassiman & Veugelers, 2002), commonly referred to as the risk of knowledge leakage (Gans & Stern, 2003) or the “paradox of openness” (Laursen & Salter, 2014). This paradox describes the fundamental tension between knowledge sharing (value creation) and knowledge protection (value appropriation) in open innovation. Open innovation may increase the imitation tendency of mimetic companies, who benefit from incurring fewer costs and inefficiencies with access to extended networks. Therefore, a company’s position on the continuum and its competitive stance in the industry depends upon its ability to remain at the technological forefront. Consequently, open innovation also poses significant governance challenges to monitoring, controlling, and managing intellectual property rights in enterprise innovation (Graham & Mowery, 2006). Hence, risk-averse companies usually have linear business models with a unilateral dependency on internal resources. This tendency to be an information hoarder lends itself to a closed innovation competitive stance. For this reason, the company’s risk strategy must also be considered, since innovation pioneers may be more risk-tolerant than those with more traditional business models. As newer, more revolutionary technologies become available, static business models with poor networks risk being on the low end of the innovation continuum. Companies that have failed to keep at the forefront of technology do not have sustainable business models and may lose their extended networks.

### Sustainable business models

**Fig. 4:**
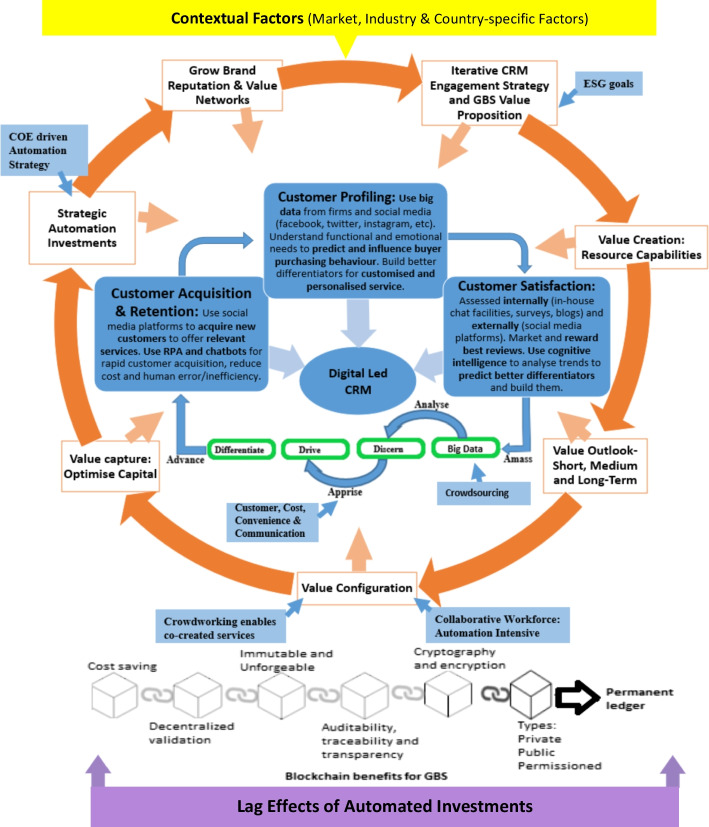
Responsive Business Innovation Model. Source: Author

The diminishing competitiveness of traditional business models (McGrath, 2010) has led to a fundamental rethinking of the firm’s value proposition for new prospects (Bock et al., 2012) on refining how an existing product or service is provided to the customer (Velu & Stiles, 2013). Reconceptualising structural elements for technology and resource capitalisation to create new activity frameworks and networks aimed at clear value propositions is known as business model innovation (Battistella et al., 2017; Hamel, 2000; Helfat et al., 2007). Therefore, business model responsiveness becomes a critical success factor in addressing challenges in the knowledge economy. A business model’s alignment and coherence should be mutually reinforcing and incorporate a response to the concomitant influence of contextual factors (Dehning & Richardson, 2002; Melville et al., 2004; Schryen, 2013) and lag effects on firm performance (Schryen, 2013). The responsive business innovation model, in Fig. 4 is a hybridisation of prior value models with interlinkages to current service technologies employed in the market, including digital platforms, crowdsourcing, blockchain, crowdworking, big data and service bots.

Figure 4 ascribes to Santos et al. (2015), where the model is more about *“how is it being done?”* than *“what is being done?* It incorporates an iterative strategy that maps cross-functional relationships between innovations and the underlying activities to be responsive to the evolving economic environment. Large corporates often use share centre services to support their network of firms under a GBS structure. However, with the evolution of AI, the GBS structure can evolve into a digital platform business model. A responsive business innovation model focuses on facilitating interactions across many shared centres by providing a governance structure and a set of standards, so that they operate as one cohesive ecosystem. It is an activity system with interconnected and interdependent activities to satisfy the market’s perceived needs (Foss & Saebi, 2018).

The responsive business innovation model enables the acquiring, developing, and integrating of key resources to overcome inertia. Introducing a new business model into an existing organisation is challenging and may require a separate organisational unit to redefine and reconfigure the model. For example, General Electric (GE) experienced business model transformation conflicts when they tried to adopt digital servitisation. There were conflicts between digital and physical service offerings, new ecosystem partnerships and traditional supply chain relationships, digital revenue and product sale models (Moazed, 2018). For this reason, positioning a Centre of Excellence (COE) is important, since it can provide the organisational structure, methodology, skills, tools and governance framework for handling the future innovation needs of a large global corporate (SSON, 2018). A GBS structure includes a COE for higher level business support and specialist work and thus is incorporated in Fig. 4. COE comprise of a centralised specialist team to promote collaboration and provide higher value services, resulting in economies of scale. COEs focus on agility,^17^ CRM and talent development while standardising and automating cross-function end-to-end process ownership), resulting in reducing costs and harnessing process efficiency (SSON, 2018). Examples of these are procure-to-pay (supply chain and accounting) and hire-to-retire (HR and accounting. The positioning of the GBS is better placed by groups of talent (area of expertise) rather than location, function or lowest costs.

The CRM literature provides a framework to delve into human motivations concerning their buying incentives, biases and emotional connections. For this reason, CRM is at the heart of the business model with AI differentiators (McAfee et al., 2012; Payne & Frow, 2005; Stringfellow et al., 2004) that responds to evolving consumer behaviour and expectations. The deep knowledge of consumers’ emotional and functional needs allows businesses to optimise capital to address those needs. This strategic response to customer needs and experience requires standardisation (lower costs, benchmark service quality) and differentiation (premium service). For instance, businesses could standardise business processes through RPA for efficiency gains but personalise services via service bots for market differentiation.

Service bots are key components of a digital strategy for entities searching for innovative and cost-effective means to build closer customer relationships (Artificial Solutions, 2020). With a GBS structure, the service bots may need to be multilingual due to the diversified client base. Furthermore, by integrating with social media (shown in Fig. 4), service bots can access clients’ online data and learn their preferences, sentiments, outlooks and proclivities. The data from clients’ online presence are often undervalued, but access to this enables businesses to transcend beyond basic business intelligence. Therefore, the service bot’s initial customer interaction will offer a superior service through seamless verification of personal information (similar to the Facebook sign-up process) and quick information transfer through hyperlinks. A seamless trail of conversations can be achieved whenever users swap from device to device (cross-platform^18^), since this practice improves engagement and customer fulfilment (Artificial Solutions, 2020). The increased customer engagement means more actionable and enriched data to train service bots to personalise the customer’s experience. In so doing, service bots can service customers more competently and cost-effectively without human error (Artificial Solutions, 2020; Kiat, 2017).

A limitation of service bots is that humans can notice tone and subtext in a way that a service bot could never master. This disparity calls for cross-functional collaboration between service bots and higher skilled humans, transitioning toward blended workforces. Data-centric CRM harness the potential of big data to focus on not only the functional but also the deeper psychological aspects of buying behaviour (Stringfellow et al., 2004). Access to client data is essential for value creation (Paiola & Gebauer, 2020) to improve existing services and create novel innovations (Opresnik & Taisch, 2015) within the confines of privacy laws. Automating customer interaction with service bots (see Fig. 4) allows for a higher degree of message personalisation without increasing personnel costs. In-depth analysis of unstructured conversational data conveys perceptions on what is done well or what can be improved by the business to develop market differentiators for a strategic competitive advantage. Smart analytics, such as sentiment analysis, support businesses in gauging their customers’ mindsets^19^ and analysing the customer’s journey more effectively while remaining within the confines of data safety legislation.

Strategy guides and shapes by including the company’s brand reputation, Fig. 4. The iterative CRM engagement strategy and value outlook (short, medium and long term) is built from big data collected from the AI-led CRM and crowdsourcing from their networks. This process allows companies to leverage their large network of end-users to inform the co-created products, services and experiences. A large network also provides microwork opportunities through crowd-working platforms for comprehensive support and supplement human labour. However, managing the trade-offs between stakeholders, technology, and societal benefits is important. Stakeholder engagement is essential in identifying key stakeholder requirements for these benefits to occur. Accordingly, business models should recognise and incorporate environmental, social and governance (ESG) goals, whereby trade-offs must be managed. For instance, automation disrupts the human capital leverage model, in which a trade-off exists between harmonising the prospective savings from automation and the human impact of job losses. Due to the escalation of global warming, business models must also incorporate innovative sustainable environmental solutions (Carayannis et al., 2020). Therefore, innovations must be expanded beyond service innovations to ESG innovations.

In Fig. 4, the benefits of using blockchain technology in a business model are also presented. Blockchain represents an endlessly accumulating list of records stored in “blocks” protected using cryptography principles (Arnaut & Bećirović, 2020). The peer-to-peer protocol ensures unambiguous and common ordering of all transactions in blocks, a process that guarantees consistency, decentralisation, integrity and auditability (Arnaut & Bećirović, 2020; Yuan & Wang, 2018). These features make the blockchain’s permanent ledger resistant to data manipulation, which is a value contribution to the company.

### Value creation

**Fig. 5:**
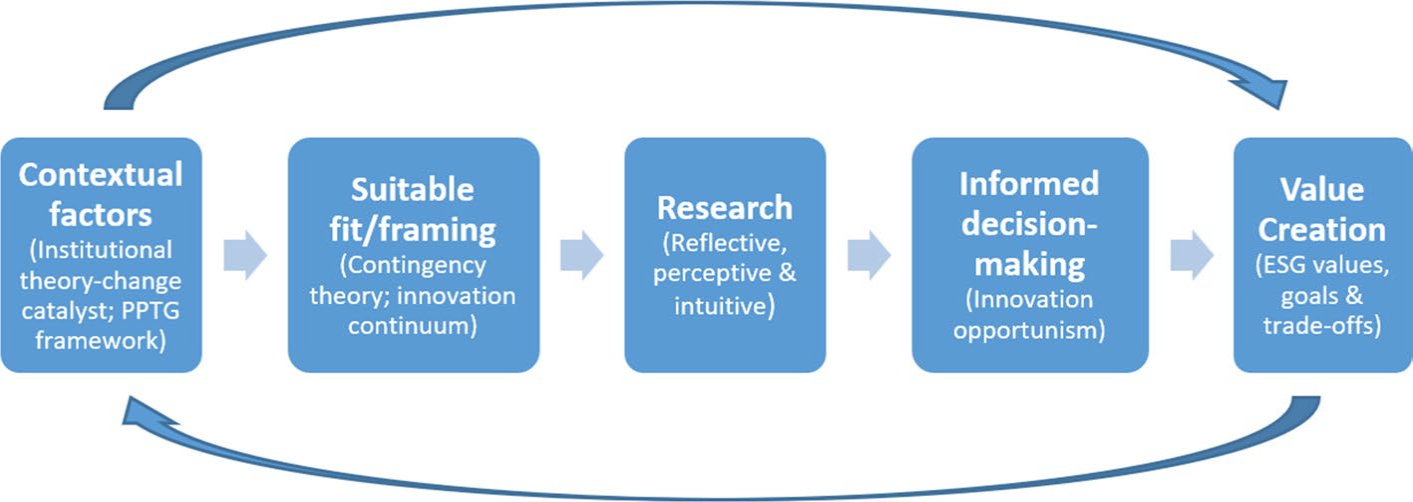
Value creation cycle. Source: Author

A business model’s lifecycle involves *“periods of specification, refinement, adaptation, revision and reformulation”* (Morris et al., 2005 pg.732). The business model’s initial period in the lifecycle has a process of trial and error, where core decision-making delimits the firm’s evolution. For this reason, a value creation cycle is essential to harness a sustainable competitive advantage by continuously refining, adapting, revising and reformulating a business model to counteract the limitation of becoming static. In Fig. 5, the importance of the continual assessment of the contextual factors, and the suitability thereof, feed into the value creation cycle necessitating the need for change. However, the suitability of this change must be assessed in terms of the company’s contingencies. Research is necessary for informed decision-making on whether the change is incremental versus transformative to reap all the benefits and value that innovations offer. For value creation, the decision-making process should be free from bias and consider the business’s ESG values, goals, and trade-offs. It is also important to be cognisant that there is a time lag before benefits can be realised. A value architecture may also assist in alleviating some of the trade-offs, particularly structuring a digitalisation committee.

**Fig. 6:**
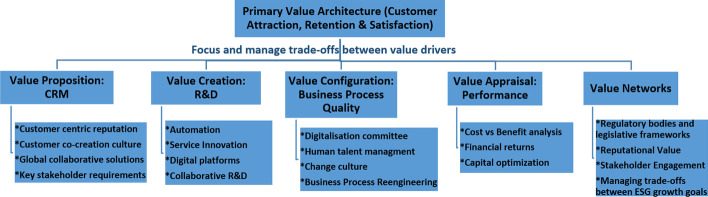
Value Architecture. Source: Author

The value architecture (Osterwalder & Pigneur, 2010), presented in Fig. 6, allows a responsive business innovation model to capture and create market activation to build the deep, compelling experiences customers desire with service-related products. However, there is a need to balance the trade-offs between conflicting value drivers. For instance, costly R&D may have environmental consequences that conflict with the desire to provide a good return on capital. For this reason, a clear value preposition is the first step in the value architecture. A value preposition is the underlying economic logic explaining how value is delivered to customers at the appropriate cost (Magretta, 2002). The building blocks of value proposition, configuration, delivery and capture (Osterwalder & Pigneur, 2010; Osterwalder et al., 2005) must be considered to develop a sustainable competitive advantage for the organisation (Teece, 2010). While the value preposition remains customer centred, the value configuration and capture are focused on relational selling using technological innovations. While the value delivery is focused on efficiency and service optimisation using service innovations.

## Conclusion

With the global environment moving so swiftly, multidisciplinary research is necessary to condense and intensify business knowledge. This study highlights the need to examine the discontinuous shift in the scope and culture of business models by exploring interdisciplinary streams of literature. An analysis of the recent literature revealed a lack of research fusing automated technologies in the business models of CRM-intensive companies. This study bridged the theoretical frameworks of organisational theories to learn how contingent characteristics influence the design and function of business models. A key contribution was the inclusion of structural elements (GBS, CRM and AI) to design a responsive business innovation model to create, deliver and capture value. It was established that AI-led CRM in a GBS structure yields a greater focus on generating innovative services that satisfy customers’ emerging needs as well as balance ESG goals. Instead of just customers just being consumers, they can be strategic networks to collaborate and co-create outcomes by integrating CRM and AI technologies into a GBS structure.

Global businesses must update their cost focussed models to transcend into the digital age by moving forward on the innovation continuum model and refocussing on customer-centric service innovations to thrive in this evolving environment. An over-reliance on past successful formulae and static business models leads to the eventual demise of AI-complacent companies. A prime example was seen during the COVID-19 pandemic when some businesses adapted swiftly to the enforced lockdowns using more digital avenues of earning revenue, while others failed to advance up the innovation continuum and closed their businesses, resulting in the loss of millions of jobs. The COVID-19 pandemic is not the only crisis faced by the global economy, since there have been other life-threatening epidemics, such as the Zika virus, MERS, Swine flu, SARS, Aids and Ebola. Businesses need to adapt to the ever-changing environment with cognitive flexibility and agility to transform their business in the wake of any crisis. Structures such as the COE may assist companies in averting the risk of unconscious incompetence in respect of evolving AI and place them at the forefront of the innovation continuum for sustained viability. Static business models can use existing digital platforms to enhance their services, enabling them to move up the innovation continuum. These businesses will have collaboration and co-creation opportunities from the large networks on the high end of the innovation continuum.

This article illustrated the benefits of AI, specifically how service bots can assist in creating new and improved business models in business-to-business and business-to-consumer markets with CRM adoption. Since service bots are a market differentiator, businesses at the forefront of service innovation are assured of resilience, even when faced with the threat of a pandemic. Service bots use real-time data to predict and influence customer behaviour, preferences, buying incentives, and spending tendencies. The un-leveraging of the human capital model has accelerated at an unprecedented level amid the COVID-19 pandemic and is foreseen as being at its most impactful in the post-pandemic period. The effects of AI technology on the human capital leverage model vary depending upon humans’ skills set. AI technology is negatively associated with low-skilled workers but significantly positively influences highly skilled workers.

Multinationals have better opportunities than single-country competitors to experiment with various business models in different geographies and then transfer those validated models to all geographies in which they can capture value (Teece, 2014). In the digital transformation era, customer-centricity and global marketplace competition, shared services have evolved from outsourcing to in-housing/re-shoring a GBS model for developing a single and consistent approach to providing internal customer services across functions and geographies. For GBS to stay at the forefront of service delivery development and remain competitive, GBS leaders must leverage and scale these new technologies. GBS’s global reach and governance, standardised processes, extended business process ownership and use of consistent operating models and technologies make them ideal candidates for implementing and delivering the aforementioned AI arbitrage benefits for their operations. This study has illustrated the tremendous strides made in AI technologies, whereby AI investment does not comprise resource-depleting disbursements but encompasses intangible assets through which the system autonomously learns and continually advances. These digital avenues provide key market differentiators in customer service.

Management cannot rely exclusively on in-house expertise and needs the benefits of mechanisms, such as crowdsourcing and crowdworking, to create a comprehensive sustainable business model. However, regulators need to be wary of the potential ramifications of crowdsourcing and crowdworking, since opportunistic companies may exploit these platforms for cheap labour. Blockchain must be considered when proposing disruptive models due to its revolutionary potential. As businesses move to scale their digital ingenuities, a focus is placed on the agility to respond to consumers’ evolving tastes with diminishing lag times due to the availability of real-time data.

Inevitable changes in business models are necessary as organisations shift how they create, capture and deliver value. For these reasons, this study developed key value drivers grounded in the theoretical framework. The key findings of this article are the various conflicting trade-offs between value drivers and ESG goals in digital business models that require executives to harmonise. Some examples of these trade-offs were:

- the societal impacts of human job losses conflict with the efficiency and cost benefits of cognitive automation,
- the utilisation of customer conversational data conflicts with remaining within the confines of data protection legislature,
- the cost of software intrusion detection systems to avoid losing confidential data conflicts with the desire to maintain profitability margins,
- the cost of innovation R&D conflicts with the desire to provide a good return on capital, and
- the standardisation of processes conflicts with the customisation of services to avoid the loss of strategic competitive advantage.

This study identified governance as a key mechanism in managing ethical issues and risks. Concerns about consumer privacy may cause governments to prevent some important innovative developments in global services (World Trade Organization, 2019). Data security is a crucial concern for any business due to security risks when handling customers’ personal information. For example, in 2018, Facebook was guilty of invading users’ personal data and giving this information to other large corporations, such as Amazon, Microsoft and Spotify, to increase Facebook’s users and revenue (Dance et al., 2018). Although regulatory user protection laws exist, businesses must employ centralised data management with cognitive analytics capabilities, encryptions, independent security audits and codes of practice. Personal identifiable data is a highly valuable commodity in the digital age but is also unsafe, since any data breaches will result in customers losing trust. Kelley (2019) recommends that a successful security protocol is to program service bots to identify personal and/or sensitive information and treat it accordingly. Systems must be able to anonymise or pseudonymise conversational data, replacing identifiable data with placeholders, so users can still understand the intent for analytics purposes but not know the customers’ identity (Kelley, 2019). Despite the challenges of surveillance and privacy issues, digital technologies are increasingly central to people, organisations and societies (Flyverbom et al., 2019). For instance, the UK government has invested more than £1 billion into an AI industrial strategy (Berditchevskaia & Baeck, 2020), thus, illustrating that some countries have grasped the opportunity to build value-added resiliency into a service delivery model.

## Recommendations

Companies should be aware of their business model lifecycle to avoid becoming stagnant. Therefore, it is recommended that they adopt a responsive business innovation model with a value-creating cycle to continuously refine, adapt, revise and reformulate their business model. To achieve this, companies should also have an innovation strategy driving a customer-centric service innovation culture while reducing costs and leveraging the finest skills. Organisations should consider establishing a COE with an innovation leader to be at the forefront of innovative technologies.

The COE would seize, assess and manage cognitive automation technologies for data governance. The COE is vital for providing leadership, driving change, and influencing business strategy and multiple onboard stakeholders across the business. COE’s essential function is driving an automation strategy as follows:

- Develop an iterative strategy to extend and expand existing capabilities through automation.
- Drive a holistic AI-enabled disruptive operating model, similar to the model proposed in this article, that is cost-efficient and leverages ‘fit-for-purpose’ technology to inspire ‘out-of-the-box’ thinking and nurture an entrepreneurial ethos.
- Incorporate and harness a digital platform strategy management that accelerates the rate of digital platforms to realise cost savings and drive resiliency.
- Initiate regular consolidating and mapping of business processes to identify areas of duplication and labour-intensive processes for an automation analysis to appraise potential benefits.
- Create an AI-intensive GBS with an effective COE to use cognitive automation technologies in customer-centric service delivery.
- Ensure CRM focuses on new customer onboarding forms and data-driven methods.
- Benchmark against industry and competitors to ensure that the company’s technology has a competitive advantage.
- Create consistent and frequent communication channels between COE and those charged with firm governance.
- Design a data governance model to determine control and direct the use of data (how and for what purpose).
- Create guidelines on data protection, privacy, intellectual property rights and ethical issues in data management.

It is also highly recommended that the public sector employs AI-intensive technologies, specifically RPA and service bots, that can streamline business processes. This sector’s work is extremely labour intensive, which is inefficient and resources depleting, given the recent rise in digital technologies. The large burden placed on taxpayers to supplement the ever-increasing public sector budgets is not met with improved outcomes. Lower level public officials’ mundane and repetitive work, such as capturing information from one system to another, using ineffective reporting templates and manual month-end tasks, are time-consuming, costly and widen the margin for human error. The public sector is also continuously dealing with fraud, tender bribes and schemes that impair its ability to deliver public services efficiently. The employment of digital agents can improve and expedite these laborious, inefficient and frustrating processes and, even more importantly, alleviate fraud to some degree.

## Future research agenda

This study focused on the value of service innovation technologies in responsive business innovation models. However, there is an abundance of future research explorations in the list below, which is not exhaustive.

### Service bots

- Research that empirically tests the customers’ satisfaction journey with digital workers versus human workers, particularly from a customer demographic perspective. For instance, NLP has made strides in making service bots more humanlike. However, there needs to be research that interrogates which customer demographics are more amenable to service bot services and which are not. Furthermore, research needs to be conducted on service bots’ ability to match their customers’ evolving needs.
- There should also be studies examining the emotional consequences on customers when their needs are addressed by service bots, particularly from a customer demographic perspective and any potential extensions to service bot biases.
- Research examining customers’ concerns over privacy and data leakages and which service bot interactions are more likely to trigger these concerns.
- Investigations into the potential impact on the company reputation/brand when faced with negative service bot interactions and biases, amongst others.
- Research investigating potential trust or control issues when customers and employees rely on work performed by service bots.

### Public service sectors

- As with institutional theory, government intervention is also necessary for a functional digital ecosystem concerning infrastructure and access to funding and investment resources. Studies should investigate government funding structures to encourage more innovative R&D.
- An appraisal of the public sector’s readiness for the digital transformation of their business model, since automated processes will result in societal benefits of service efficiency and tax savings for citizens.
- An empirical study on the suitability of a GBS innovation model for the external audit service. Due to the nature of their service, there is potential for a suitable fit.

### Open source/collaborative technologies

- An investigation into the use of open innovation systems and collaborative platforms in assisting start-up companies with their digital transformation.

## Data Availability

Freely available using online research databases.

## Abbreviations

AI: Artificial intelligence
CIO: Chief information officer
COE: Centre of excellence
COVID-19: Coronavirus disease 2019
CRM: Customer relationship management
ERP: Enterprise resource planning
ESG: Environmental, social and governance
GBS: Global business services
IT: Information technology
OECD: Organisation for economic co-operation and development
NLU: Natural language understanding
NLG: Natural language generation
NLP: Natural language processing
RPA: Robotic process automation
R&D: Research and development
Service Bot: Service robot
Softbots: Software robots

## Footnotes

1 Digitalisation or digital transformation is the use of AI technology in the business processes and activities of a company.

2 AI is distinct from conventional information technology and is defined as the ability to learn, connect, assimilate and exhibit human intelligence.

3 Automation is defined as the employment of technologies to perform a process or task that reduces human intervention.

4 Innovation in terms of this research refers to business model, service and technological innovation.

5 A value proposition enables stakeholders to understand how the business intends to use its strategic resources, which is then mapped to the business model.

6 The transition from products and add-on services to smart solutions with connectivity, monitoring, control, optimisation and autonomy is known as digital servitisation.

7 The knowledge economy is defined as production and services based on knowledge-intensive activities that contribute to an accelerated pace of technological and scientific advance as well as equally rapid obsolescence (Powell & Snellman 2004 pg. 201).

8 Wisdom of crowds uses a wide range of annotators to create large datasets, for example, Wikipedia.

9 Open innovation is the free flow of knowledge to accelerate internal and external innovation.

10 Crowdsource is an open call to internet users to get innovative solutions.

11 Crowdwork is “the performance of tasks online by distributed crowd workers who are financially compensated by requesters (individuals, groups, or organizations)” (Kittur et al., 2013 pg. 1).

12 Business processes are activities that underly value-generating processes such as transforming inputs to outputs (Melville et al., 2004).

13 Machine learning allows a machine to learn by using algorithms to analyse and draw inferences from patterns in data without direct intervention.

14 NLU helps bots understand the user by using language objects (such as lexicons, synonyms and themes) in conjunction with algorithms or rules to construct dialogue flows that tell the chatbot how to respond.

15 NLG enables bots to interrogate data repositories, including integrated back-end systems and third-party databases for information to be used to create meaningful and personalised responses that are beyond pre-scripted responses.

16 Intelligent malware is AI-based and exploits vulnerabilities by mimicking normal user behaviour to avoid being detected.

17 Agility can be described as a dynamic process of anticipating or adjusting to trends and customer needs without diverging from the company vision (Fartash et al., 2012).

18 Cross-platforms recognise inter-relationships and complimentary services through different software applications and devices.

19 Mindset is the attitudes and norms that either inhibit or encourage people’s or firms’ decisions.
